# Frailty of older patients is not reflected in the melanoma tumor microenvironment and treatment response to immune checkpoint blockade therapy

**DOI:** 10.1136/jitc-2026-015212

**Published:** 2026-09-18

**Authors:** Asli Özkan, Marieke IJsselsteijn, Ziena Abdulrahman, Manon van der Ploeg, Ellen Kapiteijn, Marije Slingerland, Els M E Verdegaal, Marij J P Welters, Sjoerd H van der Burg, Johanneke E A Portielje, Noel F C C de Miranda, Nienke A de Glas

**Affiliations:** 1 Department of Medical Oncology, Leiden University Medical Center, Leiden, The Netherlands; 2 Department of Pathology, Leiden University Medical Center, Leiden, The Netherlands; 3 Department of Medical Oncology, Oncode Institute, Leiden University Medical Center, Leiden, The Netherlands; 4 Department of Medical Oncology, Helse Førde, Førde, Norway

**Keywords:** Immune Checkpoint Inhibitor, Geriatric, Tumor microenvironment - TME

## Abstract

Older adults with frailty demonstrate systemic immunosenescence, including T-cell exhaustion and reduced immune diversity, yet maintain immune checkpoint blockade (ICB) efficacy comparable to that of younger patients. Baseline immune cell composition in both the tumor microenvironment (TME) and peripheral blood has been linked to ICB response in melanoma. To address this paradox, we investigated whether frailty is associated with alterations in the TME immune composition in melanoma.

We applied imaging mass cytometry with a 40-marker panel to comprehensively characterize the pretreatment immune TME in formalin-fixed, paraffin-embedded tissue from 23 patients with melanoma, stratified by age, frailty status (as determined by the Clinical Frailty Scale), and clinical benefit from ICB. 33 immune cell phenotypes were identified and compared across patient groups.

Frail patients exhibited significantly higher densities of CD4^+^CD45RO^+^ memory T cells compared with non-frail older patients. No other differences in the immune TME were observed between frail and non-frail patients, and overall survival was comparable between these groups. In contrast, multiple age-related differences were observed, including reduced naive T-cell densities and increased myeloid cell infiltration in older patients, consistent with features of immunologic aging. Clinical benefit from ICB was associated with greater overall immune infiltration and lower granulocyte densities, independent of age or frailty status.

Despite previously demonstrated systemic immunosenescence, the melanoma immune TME preserved in older adults with frailty, consistent with observed preservation of ICB efficacy.

## Introduction

 The aging immune system presents a paradox in cancer immunotherapy. Older adults exhibit diminished immune responses to vaccination and show hallmarks of immunosenescence, including T-cell exhaustion and reduced repertoire diversity.^1 2^ Frailty, an aging-related syndrome of decreased physiological reserve and increased vulnerability to stressors, is associated with further reductions in vaccine effectiveness and adverse health outcomes.^3 4^ Given these systemic immune deficits, one might expect diminished efficacy of immune checkpoint blockade (ICB) therapy. Yet paradoxically, clinical evidence demonstrates that ICB efficacy in older patients, including those with frailty, is comparable to that observed in younger patients.^5–8^

Despite this preserved efficacy, ICB therapy benefits only up to 50% of patients and is associated with immune-related adverse events that carry greater consequences for older adults with frailty.^9^ No biomarker has yet demonstrated sufficient predictive value to guide ICB use in advanced melanoma.^10^

Baseline immune cell composition in both the TME and peripheral blood has been linked to ICB response in melanoma, with the frequencies and phenotypes of various intratumoral and circulating immune cell populations predicting treatment outcomes.^11 12^ While the impact of frailty on systemic immunity is well characterized, its influence on the immune composition of the tumor microenvironment (TME) remains largely unknown.^13–15^

To address this knowledge gap, we comprehensively characterized the immune cell composition of the TME in pretreatment melanoma samples, across age groups and frailty states. By correlating TME features with clinical variables and ICB outcomes, we sought to determine whether frailty-associated systemic immune dysfunction is mirrored within the TME and to gain insight into mechanisms that may underlie sustained immunotherapy efficacy in frail older adults.

## Materials and methods

In this study, we applied imaging mass cytometry (IMC) to comprehensively characterize the TME in a cohort of patients with melanoma stratified by age, frailty and response to ICB therapy.

### Patient samples

Tumor samples were obtained from patients enrolled in the prospective TCIMM study (longitudinal exploratory analysis of tumor-specific T-cell immunity and microbiome/calprotectin testing in patients with solid tumors), which included all patients treated with ICB therapy against programmed cell death 1 (PD-1) and/or cytotoxic T-lymphocyte-associated protein 4 (CTLA-4) molecules. Specifically, pretreatment formalin‐fixed paraffin‐embedded (FFPE) biopsies from patients of all ages with histologically confirmed advanced cutaneous melanoma were retrospectively included. A total of 30 patients were initially identified. Seven samples were excluded during quality control: one region of interest (ROI) was removed due to poor image quality, and six samples were excluded due to sample-level technical issues. The final cohort comprised 23 patients with evaluable IMC data.

### Clinical frailty and response evaluation

In this study, frailty was retrospectively assessed by a clinician using the Clinical Frailty Scale (CFS) V.2.0, a validated 9-point scale with established reliability for retrospective use.^16^ Patients with a CFS score of 0–3 were classified as non-frail, while higher scores indicated increasing frailty and associated risks.^17^ Patients with CFS ≥4 were classified as frail, consistent with prior studies demonstrating worse outcomes at this threshold.^18 19^ Since frailty rarely occurs in younger individuals, all frail patients in our cohort were aged ≥65 years. Consequently, non-frail patients were analyzed as a combined group regardless of age for the primary frailty status comparison.

Treatment response was evaluated at 3 and 6 months as per standard of care, using CT, positron emission tomography-CT, and/or MRI imaging. Response was evaluated according to the Response Evaluation Criteria in Solid Tumors V.1.1. Clinical benefit was defined as complete or partial response, or stable disease at 6 months. Progression at either time point indicated no clinical benefit. Patients were followed for up to 2 years or until death.

### Imaging mass cytometry

For this study, a 40-marker panel was developed, and IMC was used to provide detailed spatial and phenotypic characterization of immune cell populations in pretreatment FFPE melanoma tissue sections. The complete panel can be found in online supplemental table 1. The detailed methodological protocol has been previously described in published studies.^20 21^

FFPE melanoma tissue blocks were sectioned into 4 μm thick slices and mounted on silane-coated glass slides (VWR, Radnor, Pennsylvania, USA). A dermatopathologist annotated tumor regions on H&E-stained slides, blinded to clinical outcomes and patient characteristics. These annotated H&E slides were used as references for spatial mapping in downstream IMC analyses. Depending on tumor size, 1–3 ROIs of 1×1 mm were analyzed per patient. To ensure consistent interpretation across samples, image data were normalized using Penguin.^22^ Phenotypes per image were extracted to calculate the mean cell density per mm^2^ for every sample. High thresholds were required due to background signal and interference from melanin-positive cells across several channels. Cell segmentation was performed with Mesmer, enabling transformation of multiplexed images into single-cell protein expression data.^23^ To identify major cell populations from the 40-marker panel, dimensionality reduction and clustering techniques were applied. Specifically, iterative FlowSOM clustering was used to classify cells into lymphoid, myeloid, stromal, and epithelial/melanocyte categories.^24^ Identification of tumor cells in melanoma tissues was inherently challenging due to lack of a dedicated melanoma tumor cell marker and phenotypic overlap with melanocytic populations. An overview of the cell type lineage markers for cell type identification and clustering levels of the cell phenotypes are provided in online supplemental table 2 and 3. This clustering of cell phenotypes was performed independently of patient clinical characteristics, treatment regimens, or response outcomes, using only immunological marker expression profiles to define biologically relevant cell populations. Processed IMC data were visualized using custom R scripts. Data processing and visualization used the tidyverse package collection (V.2.0.0). Heatmaps were generated using ComplexHeatmap (V.2.20.0).

### Statistical analysis

Patients were stratified by frailty status and by age (<65 years vs ≥65 years), with ≥65 years as the primary cut-off for consistency with the 2023 American Society of Clinical Oncology (ASCO) Geriatric Oncology Guideline^25^ and were additionally grouped according to clinical response to ICB therapy. Comparisons between groups were performed using Fisher’s exact test. The densities and proportions of cell phenotypes were compared between non-frail and frail, young and old, and responder and non-responder groups. For these analyses, we used the intermediate phenotype classification to balance phenotypic specificity with statistical power. Comparisons of cell densities and proportions between groups were conducted using Wilcoxon rank-sum tests. To reduce multiple testing burden and focus on biologically meaningful comparisons, statistical testing was performed only for cell populations demonstrating observable differences between groups and relevance to ICB response mechanisms. Adjusted p value for multiple testing was calculated using Benjamini-Hochberg correction. Survival analyses were conducted using Kaplan-Meier curves and log-rank tests (survival V.3.7.0, survminer V.0.5.0). Statistical significance was defined as p<0.05. Statistical analyses were performed using R V.4.5.0 (R Foundation for Statistical Computing, Vienna, Austria).

## Results

### Patient cohort characteristics

**Figure 1:**
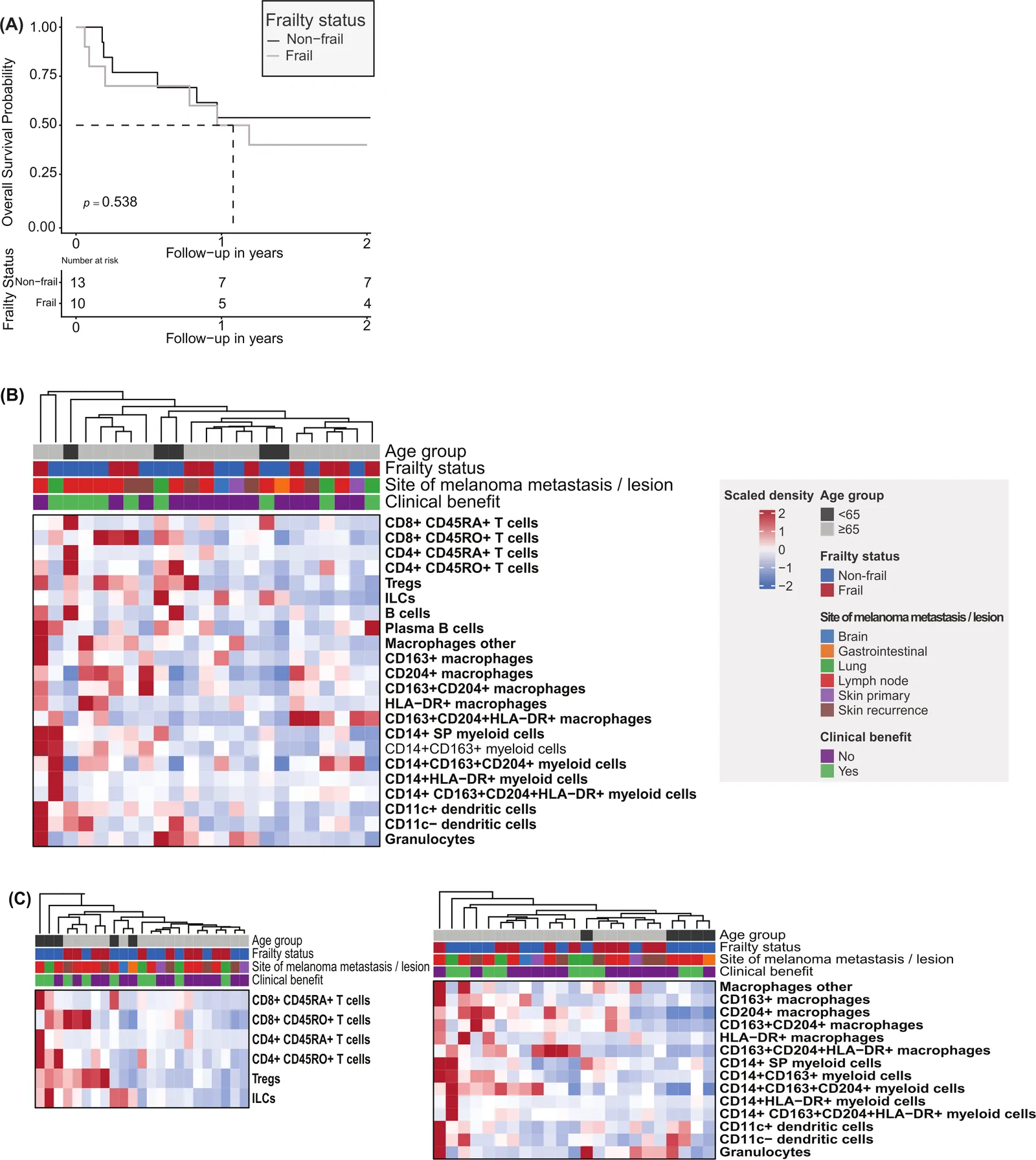
Frailty status does not associate with survival or tumor immune composition in patients with melanoma. (**A**) Kaplan-Meier curves of overall survival in non-frail versus frail patients with melanoma. (**B**) Heatmap presenting the unsupervised clustering of untreated melanoma tumor samples. The heatmap displays the mean cell density Z-score of the identified cell phenotypes, excluding sarcoma and epithelial cells. The samples are annotated for age group, frailty status, anatomical location of the melanoma sample, and whether the patient had clinical benefit from immunotherapy. (**C**) Heatmaps showing unsupervised hierarchical clustering of treatment-naive melanoma samples based on mean cell density Z-scores of T cell, ILC, and myeloid cell phenotypes. Samples are annotated by patient age group, frailty status, tumor anatomical location, and clinical benefit from immunotherapy. ILC, innate lymphoid cell. Tregs, regulatory T cell.

A total of 23 patients with advanced cutaneous melanoma were included in the study (table 1), comprising 18 patients (78.3%) aged ≥65 years and 5 patients (21.7%) aged <65 years. Using the CFS, 13 patients (56.5%) were classified as non-frail (CFS 0–3) and 10 patients (43.5%) as frail (CFS ≥4). No patients in the cohort were classified as both young and frail. Melanoma samples were obtained from diverse anatomical sites. The most common biopsy locations were lymph nodes (43.5%), followed by skin (30.4%) and lung (17.4%). Less frequent sites included primary brain (4.3%) and gastrointestinal tract (4.3%). The majority of patients (73.9%, n=17) received first-line anti-PD-1 monotherapy (nivolumab or pembrolizumab) as ICB treatment, while six patients (26.1%) received combination therapy with anti-PD-1 and anti-CTLA-4 (ipilimumab). All patients treated with combination therapy were ≥65 years of age. Clinical benefit rates were comparable between regimens (combination therapy 2/6, 33.3%; monotherapy 7/17, 41.2%). When grouped by frailty status, clinical benefit was observed in 46.2% (6/13) of non-frail patients and 30.0% (3/10) of frail patients, with no significant difference between groups (OR 0.52, 95% CI 0.06 to 3.73, p=0.670). Consistent with these findings, overall survival did not differ significantly between non-frail and frail patients (p=0.538) (figure 1A).

**Table 1:** Patient, tumor, and treatment characteristics

Variables		N (%)
Age group	<65 ≥65	5 (21.7) 18 (78.3)
Frailty status	Non-frail Frail	13 (56.5) 10 (43.5)
Tumor location	Brain Lung Lymph nodes Gastrointestinal tract Skin	1 (4.3) 4 (17.4) 10 (43.5) 1 (4.3) 7 (30.4)
Treatment type	Anti-PD-1 Anti-PD-1/anti-CTLA-4	17 (73.9) 6 (26.1)

CTLA-4, cytotoxic T-lymphocyte-associated protein 4; PD-1, programmed cell death.

### TME composition according to age and frailty status

IMC was successfully performed on 38 ROIs from 23 melanoma samples. Unsupervised clustering analysis of the 40-marker IMC panel identified 33 immune cell phenotypes (online supplemental table 3). These detailed phenotypes were then grouped into 22 intermediate phenotypes and three major categories based on the shared phenotypic/lineage markers (T cell/ILC, B cell, and myeloid lineages, online supplemental table 3). This hierarchical classification approach enabled comprehensive profiling of TME immune composition at multiple levels across patient subgroups defined by frailty status, age, tumor location, and clinical benefit from ICB (figure 1B, online supplemental figure 1). Representative IMC images were generated to visualize spatial immune cell patterns across age, frailty, and treatment response groups (online supplemental figure 1).

A comprehensive heatmap of all identified cell populations is provided in online supplemental figure 2. Stromal and vascular compartments did not differ significantly across age, frailty, or response groups.

Before examining frailty-specific differences in the TME, we first confirmed that our IMC approach accurately recapitulated well-established age-related immunological patterns, thereby providing methodological validation. In general, younger patients displayed a higher abundance of T-cell subsets (figure 1C, online supplemental figure 3). Consistent with the expected increased abundance of naïve T cells in younger individuals, younger patients exhibited higher densities of CD4^+^CD45RA^+^ T cells compared with older patients (median: 174.0 vs 35.4 cells/mm²), although not statistically significant (p=0.117) (online supplemental figure 3). Although it is possible that a fraction of this population represents terminally differentiated effector memory (TEMRA) T cells, the strong association of this subset with younger patients supports its predominant classification as naïve T cells. To more directly address differentiated and senescent CD8^+^ T cells, we examined CD3^+^CD8^+^CD27^−^CD57^+^ T cells as a surrogate marker for this population (online supplemental figure 4). Median frequencies were consistently higher in the older/frail group across all three comparisons: 0.99% versus 2.10% by age (≥65 cut-off; p=0.180), 1.01% versus 2.22% by age (≥70 cut-off; p=0.197), and 0.99% versus 2.10% by frailty status (p=0.226), consistent with the expected accumulation of senescent CD8^+^ T cells with aging and frailty.

On the other hand, older patients exhibited elevated myeloid cell densities, particularly CD14^+^CD163^+^CD204^+^ myeloid cells (p=0.023), consistent with age-related increases in myeloid-derived suppressor cells and pro-inflammatory myeloid populations (figure 1C, online supplemental figure 5).^26 27^

To address our primary research question, we compared TME immune cell densities and phenotypic distributions between non-frail and frail patients. Unsupervised hierarchical clustering analysis at both the intermediate level (figure 1B) and detailed phenotype level (online supplemental figure 2) revealed no distinct separation of samples based on frailty status. Quantitative statistical comparison confirmed these findings: none of the 3 vascular, 5 stromal, 1 epithelial, 1 melanocyte, 33 detailed immune cell phenotypes, or 22 intermediate immune cell categories showed statistically significant differences between non-frail and frail groups after Wilcoxon rank-sum tests with Benjamini-Hochberg (BH) false discovery rate correction for multiple testing. To determine whether frailty relates to TME composition independent of chronological age, we compared immune cell densities between frail and non-frail patients within the older cohort (n=18, all ≥65 years) (online supplemental figure 6A). Frail older patients demonstrated significantly higher densities of CD4^+^CD45RO^+^ memory T cells compared with non-frail older patients (median: 202.5 vs 41.5 cells/mm², Benjamini-Hochberg (BH)-adjusted p=0.012) (online supplemental figure 6B). No other immune populations differed significantly between frailty groups after correction for multiple testing. Because CD4^+^CD45RO^+^ memory T cells were the only lymphocyte population that differed significantly between groups and have a well-established role in regulating myeloid and B-cell function, we performed correlation analyses between CD4^+^CD45RO^+^ memory T-cell density and two B-cell and fourteen myeloid populations (online supplemental figure 7). While CD4^+^CD45RO^+^ density was positively associated with B-cell density (ρ=0.66, BH-adjusted p=0.006) and CD11c^−^ dendritic cells (ρ=0.65, BH-adjusted p=0.006), other populations showed no clear association. Patients with clinical benefit from ICB therapy demonstrated greater overall immune cell infiltration compared with non-responders (p=0.035; figure 1B and online supplemental figure 8). Responders showed a numerically higher median density of CD8^+^CD45RO^+^HLA^−^DR^+^ T cells than non-responders (2.31 vs 0.77 cells/mm²), although not statistically significant (p=0.108) (online supplemental figure 9). Next, we investigated immunoregulatory marker expression (PD-1, ICOS, HLA-DR, CD39, CD103) across CD8^+^, CD4^+^, and regulatory T cell (Treg) subsets (online supplemental figure 10). Responders displayed a trend for a higher percentage of PD-1^+^ CD8^+^ T cells (3.07% vs 1.39%; p=0.173). Non-responders displayed significantly higher granulocyte densities within the TME (60.5 vs 34.6 cells/mm², p=0.044) (online supplemental figure 11).

### Preserved CD8^+^:Treg^+^ ratios despite age-related Treg differences

Previous research has identified age-related increases in intratumoral Tregs and decreased CD8^+^:Treg^+^ ratios as predictors of anti-PD-1 resistance in melanoma.^28^ To assess whether similar patterns were present in our cohort, we analyzed Treg densities and CD8^+^:Treg^+^ ratios across age and frailty groups. Younger patients had higher median densities (25.1 vs 8.5 cells/mm^2^) of Tregs; due to the small sample size, this was not significant (p=0.210) (online supplemental figure 12A). Although Treg density differed by age, it was unclear whether this reflected an age-related imbalance in effector and regulatory T-cell populations or a proportional increase in both. As Treg abundance typically correlates with CD8^+^ T-cell infiltration as part of homeostatic immune regulation, we calculated CD8^+^:Treg^+^ ratios to assess whether this represented a proportional increase in both populations. The median CD8^+^:Treg^+^ ratio was similar between young (11.6) and older (12.2) patients (p=0.687) (online supplemental figure 12B). Within the older cohort, CD8^+^:Treg^+^ ratios also remained balanced between non-frail (9.1) and frail (12.3) patients (p=0.597) (online supplemental figure 12C).

## Discussion

This study demonstrates that frailty status does not influence pretreatment TME immune composition in patients with melanoma. Despite frailty-associated changes in systemic immunity, comparable immune infiltration patterns were observed between frail and non-frail patients, paralleled by similar overall survival outcomes.^19^ Potential age-related differences were only reflected by increased CD14^+^CD163^+^CD204^+^ myeloid cell densities in older patients. Clinical benefit from ICB was associated with greater overall immune infiltration but lower granulocyte densities, independent of age or frailty status.

Our findings complement recent observations by Tran Van Hoi *et al*, who demonstrated that despite significant frailty-associated changes in circulating immune cells in peripheral blood, including reduced naïve CD8^+^ T cells, clinical response to immunotherapy remained comparable across age and frailty groups.^19^ This preserved TME immunity, despite systemic immunosenescence, provides support for the paradoxical maintenance of ICB efficacy in frail and older patients observed in clinical practice.^5 8 19^ One notable exception within the older cohort was the fivefold elevation in CD4^+^CD45RO^+^ memory T-cell densities observed in frail compared with non-frail older patients. While this represents a significant difference at the individual phenotype level, it did not translate into differences in clinical outcomes between frailty groups. The biological significance of this finding remains unclear. CD4^+^CD45RO^+^ memory T cells can exert both supportive and regulatory functions in antitumor immunity. In peripheral blood, no difference in circulating CD45RO^+^ cells by frailty status has been reported.^29^ The potential association between frailty and elevated CD4^+^ memory T-cell infiltration in the TME warrants further investigation. Direct paired analyses of circulating and intratumoral immune populations were not feasible, as matched peripheral blood samples were not available for the majority of patients. Prospective studies incorporating paired blood and tumor tissue specimen collection would enable a more comprehensive assessment of the association between systemic and local immune responses.

In contrast to the lack of frailty-associated differences, we observed some clear distinctions in TME composition related to age and clinical benefit, consistent with established literature. While our panel did not include markers to definitively distinguish naïve T cells from terminally differentiated effector memory cells re-expressing CD45RA (TEMRA), CD45RA^+^ cells in younger individuals are predominantly naïve T cells. These findings align with the well-documented age-related decline in naïve T-cell populations resulting from thymic involution and reduced T-cell receptor diversity.^30^ The age-related increases in myeloid cell densities are consistent with known hematopoietic skewing toward myeloid lineages during aging.^26 27^

Unlike Kugel *et al*, who reported increased intratumoral Tregs and decreased CD8^+^:Treg^+^ ratios in younger patients, with these age-related immune signatures predictive of anti-PD-1 therapy resistance, we observed higher median Treg densities in younger patients but preserved CD8^+^:Treg^+^ ratios across age groups.^28^ Importantly, CD8^+^:Treg^+^ ratios remained balanced between non-frail and frail older patients. The preserved CD8^+^:Treg^+^ ratio across both age and frailty groups parallels the maintained clinical efficacy observed across these groups, suggesting that preservation of this immune balance may be a determinant of ICB response independent of age or frailty status.

The association between clinical benefit and greater immune infiltration confirms extensive literature demonstrating that “hot” immune-inflamed tumors respond more favorably to ICB. Responders exhibited significantly higher densities of tumor-infiltrating immune cells. While responders showed higher percentages of PD-1^+^ CD8^+^ T cells, this difference did not reach statistical significance. This may in part reflect technical limitations of IMC for detecting low-abundance markers such as PD-1. The elevated granulocyte densities in non-responders align with previous research linking granulocytic infiltration, particularly neutrophils, to immunosuppression and ICB resistance across multiple cancer types.^31^

### Strengths and limitations

Our study’s major strength is being the first to apply in-depth spatial quantification of the pretreatment melanoma TME specifically examining frailty-related differences. The detection of expected age-related immune patterns serves as internal validation of our methodological approach.

Several limitations warrant consideration. The small cohort size, while sufficient to demonstrate preserved TME composition across frailty states, limits statistical power for detecting subtle differences and restricts subgroup analyses. Frailty assessment was performed retrospectively using the CFS; prospective assessment using comprehensive geriatric evaluation would provide more robust characterization. We adopted ≥65 years as the primary cut-off for consistency with the 2023 ASCO Geriatric Oncology Guideline.^25^ Of note, definitions of “older” age in oncology are heterogeneous and lack a clear biological basis.^32^

Melanoma samples were obtained from diverse anatomical sites, predominantly lymph node metastases, which may introduce heterogeneity in baseline immune density. However, anatomical sites were approximately equally distributed across comparison groups, mitigating systematic bias. Patients received heterogeneous ICB regimens. In this cohort, combination therapy was administered exclusively to older patients (all ≥65), so treatment regimen is partly confounded with age. Although this does not impact the analyses of the TME with respect to age and frailty, it may affect treatment outcome analyses. Our IMC panel lacked markers to identify specific subpopulations of T cells. There were also technical limitations including melanin interference and elevated background staining that reduced sensitivity for certain populations. Finally, our findings are derived exclusively from melanoma, a highly immunogenic tumor type, and may not generalize to tumors with sparse immune infiltration.

In conclusion, despite previously demonstrated systemic immunosenescence associated with frailty, the melanoma TME maintains preserved immune composition and ICB efficacy in frail older patients. Immunotherapy decisions for frail older patients should be guided by tumor immune characteristics and clinical parameters such as lactate dehydrogenase, location and number of metastases rather than frailty status alone.

## Supplementary material

10.1136/jitc-2026-015212 online supplemental file 1

## Data Availability

Data are available upon reasonable request.

## References

[1] Ciarambino T, Crispino P, Buono P (2023). Efficacy and safety of vaccinations in geriatric patients: a literature review. Vaccines (Basel).

[2] Goodwin K, Viboud C, Simonsen L (2006). Antibody response to influenza vaccination in the elderly: a quantitative review. Vaccine.

[3] Clegg A, Young J, Iliffe S (2013). Frailty in elderly people. The Lancet.

[4] Tang F, Hammel IS, Andrew MK (2023). Frailty reduces vaccine effectiveness against SARS-CoV-2 infection: a test-negative case control study using national VA data. J Nutr Health Aging.

[5] de Glas NA, Bastiaannet E, van den Bos F (2021). Toxicity, response and survival in older patients with metastatic melanoma treated with checkpoint inhibitors. Cancers (Basel).

[6] Özkan A, Kapiteijn E, van den Bos F (2024). Adjuvant immunotherapy in older patients with stage III and resected stage IV melanoma: Toxicity and recurrence-free survival outcomes from the Dutch melanoma treatment registry. Eur J Cancer.

[7] Elias R, Giobbie-Hurder A, McCleary NJ (2018). Efficacy of PD-1 & PD-L1 inhibitors in older adults: a meta-analysis. J Immunother Cancer.

[8] Özkan A, de Joode K, Kapiteijn E (2026). Impact of geriatric impairments on outcomes of single-agent immunotherapy in solid tumors. Int J Cancer.

[9] Larkin J, Chiarion-Sileni V, Gonzalez R (2019). Five-year survival with combined nivolumab and ipilimumab in advanced melanoma. N Engl J Med.

[10] Kluger HM, Zito CR, Turcu G (2017). PD-L1 studies across tumor types, its differential expression and predictive value in patients treated with immune checkpoint inhibitors. Clin Cancer Res.

[11] Gide TN, Quek C, Menzies AM (2019). Distinct immune cell populations define response to anti-PD-1 monotherapy and anti-PD-1/anti-CTLA-4 combined therapy. Cancer Cell.

[12] Krieg C, Nowicka M, Guglietta S (2018). High-dimensional single-cell analysis predicts response to anti-PD-1 immunotherapy. Nat Med.

[13] Hou C, Wang Z, Lu X (2024). Impact of immunosenescence and inflammaging on the effects of immune checkpoint inhibitors. *Cancer Pathog Ther*.

[14] Özkan A, de Glas NA, Portielje JEA (2024). Immunotherapy: should we worry about immunosenescence?. Ageing Cancer Res Treat.

[15] Moldoveanu D, Ramsay L, Lajoie M (2022). Spatially mapping the immune landscape of melanoma using imaging mass cytometry. Sci Immunol.

[16] Rockwood K, Theou O (2020). Using the Clinical Frailty Scale in allocating scarce health care resources. Can Geriatr J.

[17] Goede V (2023). Frailty and cancer: current perspectives on assessment and monitoring. Clin Interv Aging.

[18] Sze S, Pellicori P, Zhang J (2021). Effect of frailty on treatment, hospitalisation and death in patients with chronic heart failure. Clin Res Cardiol.

[19] Tran Van Hoi E, Santegoets SJ, Mooijaart SP (2024). Blood based immune biomarkers associated with clinical frailty scale in older patients with melanoma receiving checkpoint inhibitor immunotherapy. Immun Ageing.

[20] Ijsselsteijn ME, van der Breggen R, Farina Sarasqueta A (2019). A 40-marker panel for high dimensional characterization of cancer immune microenvironments by imaging mass cytometry. Front Immunol.

[21] Ijsselsteijn ME, de Miranda N (2025). Multidimensional profiling of cancer microenvironments in FFPE tissues by imaging mass cytometry. Methods Cell Biol.

[22] Sequeira AM, Ijsselsteijn ME, Rocha M (2024). PENGUIN: a rapid and efficient image preprocessing tool for multiplexed spatial proteomics. Comput Struct Biotechnol J.

[23] Greenwald NF, Miller G, Moen E (2022). Whole-cell segmentation of tissue images with human-level performance using large-scale data annotation and deep learning. Nat Biotechnol.

[24] Van Gassen S, Callebaut B, Van Helden MJ (2015). FlowSOM: Using self-organizing maps for visualization and interpretation of cytometry data. Cytometry A.

[25] Dale W, Klepin HD, Williams GR (2023). Practical assessment and management of vulnerabilities in older patients receiving systemic cancer therapy: ASCO guideline update. J Clin Oncol.

[26] Kovtonyuk LV, Fritsch K, Feng X (2016). Inflamm-aging of hematopoiesis, hematopoietic stem cells, and the bone marrow microenvironment. Front Immunol.

[27] Young K, Borikar S, Bell R (2016). Progressive alterations in multipotent hematopoietic progenitors underlie lymphoid cell loss in aging. J Exp Med.

[28] Kugel CH, Douglass SM, Webster MR (2018). Age correlates with response to anti-PD1, reflecting age-related differences in intratumoral effector and regulatory T-cell populations. Clin Cancer Res.

[29] Buondonno I, Sassi F, Cattaneo F (2022). Association between Immunosenescence, Mitochondrial Dysfunction and Frailty Syndrome in Older Adults. Cells.

[30] Naylor K, Li G, Vallejo AN (2005). The influence of age on T cell generation and TCR diversity. *J Immunol*.

[31] Kim IS, Gao Y, Welte T (2019). Immuno-subtyping of breast cancer reveals distinct myeloid cell profiles and immunotherapy resistance mechanisms. Nat Cell Biol.

[32] Carleton N, McAuliffe PF (2022). Are the chronological age cutoffs used in clinical oncology guidelines biologically meaningful?. Nat Rev Clin Oncol.

