# Supplementary material for: Frailty of older patients is not reflected in the melanoma tumor microenvironment and treatment response to immune checkpoint blockade therapy
Source: J Immunother Cancer. 2026 Sep 18;14(9):e015212. doi: 10.1136/jitc-2026-015212 (PMC13599837; doi:10.1136/jitc-2026-015212)
Supplement: Supplementary file 1 [file jitc-14-9-s001.docx]

**Index of supplemental information**

**Supplemental methods**

**Supplemental table 1.** Imaging mass cytometry marker panel

**Supplemental table 2.** Cell type lineage markers and final cluster hierarchy imaging mass cytometry

**Supplemental table 3.** Hierarchical grouping of the highly detailed immune cell phenotypes to the intermediate level and major categories.

**Supplemental references**

**Supplemental figure 1.** Representative images generated with imaging mass cytometry.

**Supplemental figure 2.** Heatmap presenting the unsupervised clustering of all identified cell populations in untreated melanoma tumor samples.

**Supplemental figure 3.** Comparison of naïve (CD45RA^+^) and memory (CD45RO^+^) T cell densities by age groups.

**Supplemental figure 4.** Comparison of TEMRA-like CD3^+^CD8^+^CD27^−^CD57^+^ T cell frequencies by age groups.

**Supplemental figure 5.** Comparison of subpopulations of myeloid cells by age groups.

**Supplemental figure 6.** Comparison of tumor immune composition in older melanoma patients.

**Supplemental figure 7.** Correlation analyses between CD4^+^CD45RO^+^ memory T cell density and myeloid and B-cell populations.

**Supplemental figure 8.** Comparison of the total immune cell density by clinical benefit status.

**Supplemental figure 9.** Comparison of CD8^+^CD45RO^+^HLA-DR^+^ T cells densities by clinical benefit status.

**Supplemental figure 10.** Percentage of PD-1^+^, ICOS^+^, HLA-DR^+^, CD39^+^, and CD103^+^ cells across CD8^+^, CD4^+^, and regulatory T-cell (Treg) subsets in responders versus non-responders to immunotherapy.

**Supplemental figure 11.** Comparison of granulocyte densities by clinical benefit status.

**Supplemental figure 12.** Intratumoral Treg densities and CD8^+^:Treg^+^ ratios stratified by age and frailty.

**Supplemental methods**

**Patient samples**

Patient samples were collected between November 2022 and March 2024 from five hospitals in the Netherlands: the Leiden University Medical Center (LUMC, Leiden, The Netherlands), the Haga Hospital (The Hague, The Netherlands), the Haaglanden Medisch Centrum hospital (the Hague, The Netherlands), Groene Hart Hospital (Gouda, The Netherlands), and Alrijne hospital (Leiden, The Netherlands).

**Clinical frailty and response evaluation**

The Clinical Frailty Scale (CFS) is a well-validated and frequently used tool for assessing physical function, including mobility and the ability to perform activities of daily living. It helps identify patients at risk of adverse treatment outcomes due to frailty, enabling timely preventive strategies to improve patient care.(1) Previous research has demonstrated that the CFS can also be reliably applied retrospectively.(2)

For the current study, radiological response at 3 months was categorized as complete (including disease-free), partial, stable, mixed, or progressive. Patients with a complete or partial response were classified as having clinical benefit. In case of stable disease or mixed response at 3 months, further assessment was deferred until the 6-month follow-up. At 6 months, complete or partial response continued to be categorized as a clinical benefit, and stable disease at 6 months was also classified as a clinical benefit. Patients exhibiting progression at either 3 or 6 months were classified as having no clinical benefit.

**Supplemental table 1. Imaging mass cytometry panel.**

| Target | Clone | Metal | Incubation Time | Temp | Dilution |
| --- | --- | --- | --- | --- | --- |
| CD4 + 2nd AB | EPR6855 | 145 Nd | Indirect ON | 4°C | 100 |
| TCRgd + 2nd Ab | H41 | 148 Nd | Indirect ON | 4°C | 25 |
| anti-rabbit IgG | Polyclonal | 145 Nd | 1 h | RT | 100 |
| anti-mouse IgG | Polyclonal | 148 Nd | 1 h | RT | 100 |
| CD8a | D8A8Y | 146 Nd | 5 h | RT | 50 |
| PD-1 | D4W2J | 160 Gd | 5 h | RT | 50 |
| ICOS | D1K2T | 161 Dy | 5 h | RT | 50 |
| CD204 | J5HTR3 | 164 Dy | 5 h | RT | 50 |
| CD103 | EPR4166(2) | 168 Er | 5 h | RT | 50 |
| CD45RA | HI100 | 170Er | 5 h | RT | 100 |
| Caspase | 5A1E | 172 Yb | 5 h | RT | 50 |
| CD163 | D6U1J | 173 Yb | 5 h | RT | 50 |
| IFNγ | 2485E | 115 In | 5 h | RT | 100 |
| HLA-DR | TAL 1B5 | 141 Pr | 5 h | RT | 100 |
| CD11b | D6X1N | 144 Nd | 5 h | RT | 100 |
| Granzyme B | D6E9W | 150 Nd | 5 h | RT | 100 |
| CD138 | MI15 | 155 Gd | 5 h | RT | 100 |
| CD39 | EPR20627 | 157 Gd | 5 h | RT | 100 |
| VISTA | D1L2G | 158 Gd | 5 h | RT | 100 |
| CD14 | D7A2T | 163 Dy | 5 h | RT | 100 |
| CD56 | E7X9M | 167 Er | 5 h | RT | 100 |
| CD7 | EPR4242 | 174 Yb | 5 h | RT | 100 |
| CD11c | EP1347Y | 176 Yb | 5 h | RT | 100 |
| CD45 | D9M8I | 149 Sm | Overnight | 4°C | 50 |
| CD3 | EP449E | 153 Eu | Overnight | 4°C | 50 |
| PD-L1 | E1L3N | 156 Gd | Overnight | 4°C | 50 |
| FOXP3 | D608R | 159 Tb | Overnight | 4°C | 50 |
| CD27 | EPR8569 | 175 Lu | Overnight | 4°C | 50 |
| Vimentin | D21H3 | 194 Pt | Overnight | 4°C | 50 |
| Keratin | C11 and AE1/AE3 | 198 Pt | Overnight | 4°C | 50 |
| CD28 | EPR22076 | 154Sm | Overnight | 4°C | 50 |
| TGF-β | TB21 | 89 Y | Overnight | 4°C | 100 |
| CD20 | H1 | 142 Nd | Overnight | 4°C | 100 |
| CD68 | D4B9C | 143 Nd | Overnight | 4°C | 100 |
| CD31 | 89C2 | 147 Sm | Overnight | 4°C | 100 |
| CD57 | HNK-1 / Leu-7 | 151 Eu | Overnight | 4°C | 100 |
| Ki-67 | 8D5 | 152 Sm | Overnight | 4°C | 100 |
| IgG1 | EPR4417 | 154 Sm | Overnight | 4°C | 100 |
| IDO | D5J4E | 162 Dy | Overnight | 4°C | 100 |
| CD45RO | UCHL1 | 165 Ho | Overnight | 4°C | 100 |
| PDPN | D2-40 | 166 Er | Overnight | 4°C | 100 |
| CD38 | EPR4106 | 169 Tm | Overnight | 4°C | 100 |
| CD15 | MC480 | 171 Yb | Overnight | 4°C | 100 |
| β-catenin | D10A8 | 196 Pt | Overnight | 4°C | 100 |

*Abbreviations: Ab = antibody, ON = overnight, RT = room temperature.*

**Supplemental table 2. Cell type lineage markers imaging mass cytometry.**

Lineage markers are describing the used markers for cell type identification as used in this study.

| Cell type | Lineage markers |
| --- | --- |
| Immune cells | CD45+ |
| T cells | CD3+ |
| Innate lymphoid cells | CD7+CD3- |
| B cells | CD20+ |
| Plasma B cells/Plasmablasts | CD38+ |
| Macrophages | CD68+ |
| CD14+ myeloid cells | CD14+CD68- |
| Dendritic cells | CD11c+CD68- |
| Granulocytes | CD15+ |
| Stromal cells | Vimentin+CD45- |
| Vessels | CD31+ |

**Supplemental table 3. Hierarchical grouping of the highly detailed immune cell phenotypes to the intermediate level and major categories.** Epithelial cells, stromal cells, and blood vessels were excluded.

| **Highly detailed phenotypes** | **Intermediate level** | **Major groups** |
| --- | --- | --- |
| CD3^+^CD8^+^CD45RA^+^ T cells | CD8^+^CD45RA^+^ T cells | T cell and ILC lineage |
| CD3^+^CD8^+^CD45RO^+^HLADR^+^ T cells | CD8^+^CD45RO^+^ T cells |  |
| CD3^+^CD8^+^CD27^+^CD38^+^CD45RO^+^ T cells |  |  |
| CD3^+^CD8^+^CD45RO^+^ T cells |  |  |
| CD3^+^CD4^+^CD45RA^+^ T cells | CD4^+^CD45RA^+^ T cells |  |
| CD3^+^CD4^+^CD28^+^CD45RA^+^ T cells |  |  |
| CD3^+^CD4^+^CD45RO^+^ T cells | CD4^+^CD45RO^+^ T cells |  |
| CD3^+^CD4^+^CD28^+^CD45RO^+^ T cells |  |  |
| CD3^+^CD4^+^FOXP3^+^ T cells | Tregs |  |
| CD3^-^CD7^+^CD56^-^ ILCs | ILCs |  |
| CD3^-^CD7^+^CD56^+^ ILCs |  |  |
| CD20^+^ B cells | B cells | B cell lineage |
| CD20^+^HLADR^+^ B cells |  |  |
| CD20^+^CD38^+^ B cells |  |  |
| CD38^+^CD138^-^ Plasma B cells | Plasma B cells |  |
| CD38^+^CD138^+^ Plasma B cells |  |  |
| CD68^+^ Macrophages | Macrophages other | Myeloid cell lineage |
| CD68^+^CD163^+^ Macrophages | CD163^+^ Macrophages |  |
| CD68^+^CD204^+^ Macrophages | CD204^+^ Macrophages |  |
| CD68^+^CD163^+^CD204^+^ Macrophages | CD163^+^CD204^+^ Macrophages |  |
| CD11c^+^CD68^+^CD163^+^CD204^+^ Macrophages |  |  |
| CD68^+^HLA-DR^+^ Macrophages | HLA-DR^+^ Macrophages |  |
| CD11c^+^CD68^+^CD163^+^CD204^+^HLA-DR^+^ Macrophages | CD163^+^CD204^+^HLA-DR^+^ Macrophages |  |
| CD14^+^CD68^-^ Myeloid cells | CD14^+^ Single-positive myeloid cells |  |
| CD11c^+^CD14^+^CD68^-^ Myeloid cells |  |  |
| CD14^+^CD68^-^CD163^+^ Myeloid cells | CD14^+^CD163^+^ Myeloid cells |  |
| CD14^+^CD68^-^CD163^+^CD204^+^ Myeloid cells | CD14^+^CD163^+^CD204^+^ Myeloid cells |  |
| CD14^+^CD68^-^HLADR^+^ Myeloid cells | CD14^+^HLA-DR^+^ Myeloid cells |  |
| CD14^+^CD68^-^CD163^+^CD204^+^HLA-DR^+^ Myeloid cells | CD14^+^CD163^+^CD204^+^HLA-DR^+^ Myeloid cells |  |
| CD11c^+^HLADR^+^ Dendritic cells | CD11c^+^ Dendritic cells |  |
| CD11c^+^HLADR^-^ Dendritic cells |  |  |
| HLADR^+^CD11c^-^ Dendritic cells | CD11c^-^ Dendritic cells |  |
| CD11b^+^CD15^+^ Granulocytes | Granulocytes |  |

**Supplemental figures**

**Supplemental figure 1. Representative images generated with imaging mass cytometry illustrating immune differences by frailty status, age, and treatment response.** The top row presents the CD14^+^myeloid cells and macrophages (green) and T cells (red) in non-frail young, non-frail old, and frail old patients. The middle row displays the shift in naïve (CD45RA^+^, green) and memory (CD45RO^+^, blue) T cell populations across the same group. The bottom row shows granulocytes (CD11b^+^CD15^+^, green) and immune cell infiltration (CD45RA^+^ and CD45RO^+^, red) in responders versus non-responders.

**Supplemental figure 2**. **Heatmap presenting the unsupervised clustering of all identified cell populations in untreated melanoma tumor samples.** The heatmap displays the mean cell density Z-score for each identified highly detailed cell phenotype. Samples are annotated for age group, frailty status, anatomical location of the melanoma sample, and clinical benefit from immunotherapy.

**Supplemental figure 3. Comparison of naïve (CD45RA^+^) and memory (CD45RO^+^) T cell populations by age groups.** Individual data points are represented, with frail patients marked by triangles and non-frail patients by circles. Data points for patients with clinical benefit are colored green, while those without clinical benefit are shown in black. The boxplot shows the distribution of absolute cell density (cells/ mm²) in young (<65) versus old (≥65) patients, with the center line representing the median, the box showing the interquartile range (IQR), and whiskers extending to 1.5 times the IQR.

**Supplemental figure 4.** **Comparison of TEMRA-like CD3^+^CD8^+^CD27^−^CD57^+^ T cell frequencies by age groups.** Frail patients are shown as triangles and non-frail patients as circles. The boxplots show the distribution of TEMRA-like CD3^+^CD8^+^CD27^−^CD57^+^ T cell frequencies in young versus old patients. The center line represents the median, the box the interquartile range (IQR), and whiskers extending to 1.5 times the IQR.

**Supplemental figure 5. Comparison of subpopulations of myeloid cells by age groups.** Individual data points are represented, with frail patients marked by triangles and non-frail patients by circles. Data points for patients with clinical benefit are colored green, while those without clinical benefit are shown in black. The boxplots show the distribution of absolute cell density (cells/ mm²) in young (<65) versus old (≥65) patients, with the center line representing the median, the box showing the interquartile range (IQR), and whiskers extending to 1.5 times the IQR.

**Supplemental figure 6. Comparison of tumor immune composition in older melanoma patients.**

**A.** Heatmap presenting the unsupervised clustering of untreated melanoma tumor samples from older patients (≥65 years). The heatmap displays the mean cell density Z-score of the intermediate immune cell phenotypes. The samples are annotated for frailty status, anatomical location of the melanoma sample, and clinical benefit from immunotherapy. **B.** CD4^+^ CD45RO^+^ memory T cell density in older melanoma patients (≥65 years) by frailty status. The boxplots show the distribution of absolute cell density(cells/mm²) in non-frail versus frail patients. Individual data points represent patient-level median density, colored green for patients with clinical benefit from immunotherapy and black for those without.

**Supplemental figure 7. Correlation analyses between CD4^+^CD45RO^+^ memory T cell density and myeloid and B-cell populations.** CD4^+^CD45RO^+^ density was positively associated with B-cell density (ρ = 0.66, adjusted p = 0.006) and CD11c^−^ dendritic cells (ρ = 0.65, adjusted p = 0.006). CD11c^+^ dendritic cells showed non-significant associations (ρ = 0.44, adjusted p = 0.180). Grey line represents a simple linear fit for visualization only; associations were assessed using Spearman correlation.

**Supplemental figure 8. Comparison of the total immune cell density by clinical benefit status.** Individual data points represent patient-level median cell densities; frail patients are shown as triangles and non-frail patients as circles. The boxplots show the distribution of absolute cell density (cells/ mm²) in responders versus non-responders to immunotherapy, with the center line representing the median, the box showing the interquartile range (IQR), and whiskers extending to 1.5 times the IQR.

**Supplemental figure 9. Comparison of CD8^+^CD45RO^+^HLA-DR^+^ T cells** **densities between responders and non-responders.** Individual data points represent patient-level median cell densities; frail patients are shown as triangles and non-frail patients as circles, with young patients (<65 years) colored black and older patients (≥65 years) grey. The boxplots show the distribution of absolute cell density (cells/ mm²) in responders versus non-responders to immunotherapy, with the center line representing the median, the box showing the interquartile range (IQR), and whiskers extending to 1.5 times the IQR.

**Supplemental figure 10.** **Percentage of PD-1^+^, ICOS^+^, HLA-DR^+^, CD39^+^, and CD103^+^ cells across CD8^+^, CD4^+^, and regulatory T-cell (Treg) subsets in responders versus non-responders to immunotherapy.** The boxplots show the distribution of percentages of the immunoregulatory markers, with the center line representing the median, the box the interquartile range (IQR), and whiskers extending to 1.5× the IQR. Individual data points represent patient-level percentages of immunoregulatory markers among the T cells; frail patients are shown as triangles and non-frail patients as circles, with young patients (<65 years) colored black and older patients (≥65 years) grey.

**Supplemental figure 11. Comparison of granulocyte densities between responders and non-responders.** Individual data points represent patient-level median cell densities; frail patients are shown as triangles and non-frail patients as circles, with young patients (<65 years) colored black and older patients (≥65 years) grey. The boxplots show the distribution of absolute cell density (cells/ mm²) in responders versus non-responders to immunotherapy, with the center line representing the median, the box showing the interquartile range (IQR), and whiskers extending to 1.5 times the IQR.

**Supplemental figure 12.** **Intratumoral Treg densities and CD8^+^:Treg^+^ ratios stratified by age and frailty. A.** Intratumoral regulatory T-cell (Treg) densities in younger versus older patients**.** Frail patients are shown as triangles and non-frail patients as circles. **B.** CD8^+^:Treg^+^ ratios stratified by age group (young versus old). **C.** CD8^+^:Treg^+^ ratios within the older cohort, comparing non-frail and frail patients. The boxplots show the distribution of Treg cell density (cells/ mm²) and CD8:Treg ratios, in young versus old patients, with the center line representing the median, the box showing the interquartile range (IQR), and whiskers extending to 1.5 times the IQR.

**References**

1.	Subramaniam A, Ueno R, Tiruvoipati R, Srikanth V, Bailey M, Pilcher D. Comparison of the predictive ability of clinical frailty scale and hospital frailty risk score to determine long-term survival in critically ill patients: a multicentre retrospective cohort study. Crit Care. 2022;26(1):121.

2.	Davies J, Whitlock J, Gutmanis I, Kane SL. Inter-Rater Reliability of the Retrospectively Assigned Clinical Frailty Scale Score in a Geriatric Outreach Population. Can Geriatr J. 2018;21(1):1–5.
